# Efficacy and Safety of Non-Vitamin K Anticoagulants for Atrial Fibrillation in Relation to Different Renal Function Levels: A Network Meta-Analysis

**DOI:** 10.1155/2020/2683740

**Published:** 2020-04-22

**Authors:** Hao Jin, Kongbo Zhu, Lina Wang, Yifan Li, Jingjun Meng, Hong Zhi

**Affiliations:** ^1^Department of Cardiology, Affiliated ZhongDa Hospital of Southeast University (HZ), Nanjing 210009, China; ^2^Department of Epidemiology and Biostatistics, School of Public Health, Southeast University, Nanjing 210009, China

## Abstract

**Background:**

We performed a network meta-analysis (NMA) comparing the efficacy (stroke or systemic embolism) and safety (major bleeding) among different non-vitamin K antagonist oral anticoagulants (NOACs) in patients with atrial fibrillation (AF) and renal impairment, with the aim of recommending the proper drug and the dose based on renal function.

**Methods:**

We searched PubMed, EMBASE, Web of Science, and Cochrane Library with the items “dabigatran, edoxaban, apixaban, rivaroxaban, warfarin, and atrial fibrillation” through August 2019. NMA was analyzed with R (version 3.5.1, R Foundation for Statistical Computing) with the packages gemtc recalling JAGS (version 4.3.0) for the efficacy and safety of each drug with regard to different levels of renal function. NetMetaXL (version 1.6.1) and winBUGS (version 1.4.3) were used to obtain the cumulative ranking curve (SUCRA) of each drug.

**Result:**

In patients with normal renal function, dabigatran150 was ranked as the most effective drug (SUCRA 0.90), followed by dabigatran110 (SUCRA 0.68), apixaban (SUCRA 0.66), and rivaroxaban (SUCRA 0.59). With regard to the safety for preventing major bleeding, there was high probability that edoxaban30 (SUCRA 0.99) ranked first, compared to dabigatran110 (SUCRA 0.78) and edoxaban60 (SUCRA 0.66). For patients with mild renal impairment, with respect to the most effective drug for preventing stroke or systemic embolism, edoxaban60 ranked first (SUCRA 0.98), in comparison with dabigatran150 (SUCRA 0.74) and apixaban (SUCRA 0.64). Possibility of ranking first for the safest drug was edoxaban30 (SUCRA 0.99), followed by dabigatran110 (SUCRA 0.70) and apixaban (SUCRA 0.69). In patients with moderate renal function, dabigatran150 (SUCRA 0.95) ranked as the most effective drug in comparison with apixaban (SUCRA 0.66). Dabigatran110 (SUCRA 0.53), rivaroxaban (SUCRA 0.51), and edoxaban60 (SUCRA 0.50) had the similar probability of ranking third. When referred to the safest drug, probability of ranking first for preventing major bleeding was edoxaban30 (SUCRA 0.98), followed by apixaban (SUCRA 0.85) and edoxaban60 (SUCRA 0.64).

**Conclusion:**

In patients with AF and renal impairment and for patients with normal renal function, dabigatran 110 mg (bid) might have a better effect on the clinical results. And it does not coincide with patients taking dabigatran 110 mg with dose reduction for other factors including aged ≥75 years, renal impairment (CrCL 30–50 mL/min), gastritis, esophagitis, or gastroesophageal reflux, receiving concomitant verapamil, and so on. For patients with mild renal impairment, apixaban 5 mg (bid) would be a better choice for preventing stroke or systemic embolism and major bleeding, while apixaban 5 mg (bid) and edoxaban 60 mg (qd) were recommended for patients with moderate renal impairment. However, considering the fact of no RCTs for the head-to-head comparison, caution should be exercised over selecting each of NOACs for patients.

## 1. Introduction

As was shown in a recent survey published in China at the national level, the standardized overall prevalence of atrial fibrillation (AF) among the Chinese adults aged ≥40 years was 2.31% (95% CI: 2.28–2.33%). Additionally, there was a higher probability that participants with AF would have a stroke than those without AF (9.48% vs 2.26%, *P* < 0.001) [1]. According to the GARFIELD-AF registry with 2-year follow-up, the percentage (95% CI) of all-cause mortality, stroke or systemic embolism, and major bleeding (first occurrences) was 3.84 (3.68; 4.02), 1.27 (1.18; 1.38), and 0.71 (0.64, 0.79) per 100 person-years [2].

The data from the German AFNET (German Competence Network on Atrial Fibrillation) database conveyed the message that paroxysmal AF was often accompanied by chronic kidney diseases (CKD) (48.7% vs. 35.6% in CKD stages 4 and 5) and permanent AF was also more common in CKD (35.5% and 37.3% CKD stages 4 and 5), meaning that AF and CKD often coexist [3]. In patients with AF and renal impairment, the risk of 12-month recurrent stroke and death had increased in comparison to the patients with AF or CKD alone [4]. In the course of a long time, warfarin was used for the prevention of stroke of patients with AF, which had played a crucial role in anticoagulant therapy. Nevertheless, it did have some limitations for narrow therapeutic window, food interactions, necessary monitoring, and dose adjustments [5]. In light of these drawbacks, four large randomized phase III trials (RE-LY [6], ROCKET-AF [7], ARISTOTLE [8], and ENGAGE AF-TIMI 48 [9]) were performed to compare the safety and efficacy between warfarin and non-vitamin K antagonist oral anticoagulants (NOACs). The four trials had presented the results that in terms of stroke or systemic embolism, rivaroxaban, edoxaban, dabigatran 150 mg, and apixaban were noninferior to warfarin. In respect to the risk of major bleeding, rivaroxaban and dabigatran 150 mg were not associated with lower rates in comparison to warfarin, while edoxaban and apixaban tended to reduce the risk. Additionally, there was no difference between dabigatran 110 mg and warfarin. As a result, these four NOACs had been approved for the prevention of stroke or systemic and major bleeding in patients with AF and served as an alternative to vitamin K antagonists (VKAs) [10]. However, these four NOACs all had a different degree of renal elimination, with dabigatran 80%, edoxaban 50%, rivaroxaban 35%, and apixaban 27% [11]. As a consequence, the proper drug and dose should be estimated according to the renal function before the patients accept the anticoagulant therapy [12]. Recently, the meta-analysis had evaluated the efficacy and safety between the NOACs and VKA for anticoagulation in AF based on renal function. The result illustrated that NOACs would reduce the risk of stroke or systemic embolism and major bleeding in respect to different levels of renal function [13, 14]. Additionally, network meta-analysis (NMA) was also performed to compare the efficacy and safety among different NOACs, finding that apixaban would be a better choice with regard to preventing stroke or systemic embolism and major bleeding in comparison to the other NOACs [15]. However, comparing NOACS to VKA made no contributions to choosing the applicable drug for anticoagulation as the lack of head-to-head comparison in relation to renal function, including the indirect comparison without the analysis on subgroups of patients with different renal functions. One NMA just assessed the anticoagulation in patients with moderate CKD of AF [16], while paying little attention to patient with different levels of renal function. As a consequence, with the influence of different NOACs and renal function levels on anticoagulation taken into consideration to compare the efficacy and safety in patients with AF, we performed the NMA to find the proper NOAC and its dose for patients with the specific renal function.

## 2. Methods

### 2.1. Data Search

We searched the RCTs comparing the effect of NOACs preventing stroke or systemic embolism and major bleeding in patients with AF and different levels of renal function. We searched PubMed, EMBASE, Web of Science, and Cochrane Library with the items “dabigatran, edoxaban, apixaban, rivaroxaban, warfarin, and atrial fibrillation” through August 2019 (Table 1).

### 2.2. Selection Criteria

Each study was reviewed by two authors independently. When coming up with disagreements during the process, we asked for another author for help if necessary. One study included in our article had to meet the following criteria: (1) it had to be a phase III or IV randomized trial, comparing NOACs (dabigatran, rivaroxaban, apixaban, or edoxaban) with warfarin in patients with AF. (2) The patients with different renal function ought to be known and the outcome of efficacy and safety (stroke and major bleeding) should be reported based on the renal function. We selected the latest and most complete data when facing different study reporting the same trial.

### 2.3. Assessment of Risk of Bias

The bias of the study was assessed using Cochrane Collaboration Risk of Bias Tool (Higgins 2011), as follows: random sequence generation, allocation concealment, blinding of participants and personnel, blinding of outcome assessment, incomplete outcome data, selective reporting, and other bias.

### 2.4. Data Extraction

Information extracted ought to include the study design, the type of NOACs and the dose regimens, patients, median age, type of the AF, mean or median CHADS2, previous stroke, and other information about the characteristics of patients. Primary efficacy outcome was stroke or systemic embolism and primary safety outcome was major bleeding, the definitions of which were extracted from the trials. Data of the clinical outcomes was based on renal function, which was categorized using the Cockcorft-Gault formula: normal renal function (eGFR > 80 ml/min), mild renal function (eGFR > 50 and eGFR < 80 ml/min), and moderate renal function (eGFR > 30 and eGFR < 50).

### 2.5. Data Synthesis and Analysis

NMA was analyzed using R (version 3.5.1, R Foundation for Statistical Computing) with the packages gemtc recalling JAGS (version 4.3.0) based on 20,000 iterations for each four MCMC chains with a burn-in period of the initial 5,000 iterations. NetMetaXL (version 1.6.1) and winBUGS (version 1.4.3) were used to obtain the cumulative ranking curve (SUCRA) of each drug. The odds ratio (OR) and 95% confidence interval (CI) were used to analyze the efficacy and safety of the NOACs. As the efficacy and safety all were defined as bad events, OR over 1.0 and CI not containing 1.0 meant the bad effect relatively [17].

The simple ranking probability from R and packages gemtc could not offer a better suggestion for the patient to choose the proper one; as a consequence, here we calculated the SUCRA of each drug with the help of NetMetaXL and winBUGS. Larger SUCRA often meant the large tendency to achieve a better ending [18]. Although the random-effect model was more fit for our NMA than a fixed-model, it may create the poor estimates for only four RCTs included. As a result, we analyzed with both random and fixed-effect model in a Bayesian framework. The totresdev and deviance information criterion (DIC) and the residual deviance (pD) of the two models were calculated to identify the difference relatively [19]. Considering that the data of our NMA was sparse, we used informative prior to identify the heterogeneity, as was reviewed in previous article [20]. Trace plots, the Brooks-Gelman-Rubin diagnostic, and PSFR were used to ensure the convergence [21]. Taking the network plot without any loops into consideration, we found it impossible to make the evaluation of the consistency of direct and indirect comparisons.

## 3. Results

**Figure 1:**
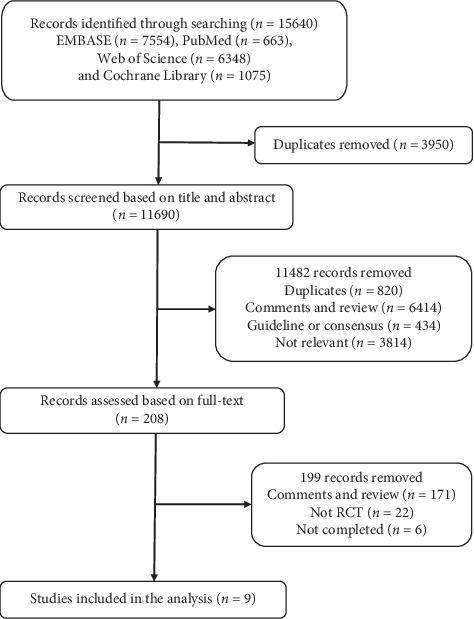
Flow diagram of the clinical trial selection process.

As is shown in Figure 1, 15640 included records were identified from EMBASE (*n* = 7554), PubMed (*n* = 663), Web of Science (*n* = 6348), and Cochrane library (*n* = 1075), and 3950 duplicates were removed. Subsequently, 11482 records were removed for not meeting the inclusion criteria and 208 records were assessed based on full-text screening. Finally, nine studies were included in our NMA identifying four phase III RCTs (RE-LY [6, 22, 23], ROCKET-AF [7], ARISTOTLE [8, 24], and ENGAGE AF-TIMI 48 [9, 25, 26]), which enrolled 71681 patients, and all the trials of low bias (Table 2). Data of the incidence of clinical outcomes was also obtained from U.S.FOOD&DRUG. The baseline characteristics of the four RCTs are shown in Table 3. In general, there seemed a little difference with respect to the age and the percentage of the women for patients participating in the trials. The four RCTs all showed high rates of paroxysmal AF in comparison to persistent or permanent AF. Patients of ROCKET-AF had a higher rate of previous VKA use, previous stroke, heart failure, and diabetes mellitus than the other trails. Besides that, patients of ROCKET-AF seemed to have a higher risk as the comparison of mean CHADS2 score between the four RCTs. Mean follow-up of the trials ranged from 1.8 to 2.8 years.

### 3.1. Bayesian Network Meta-Analysis

**Figure 2:**
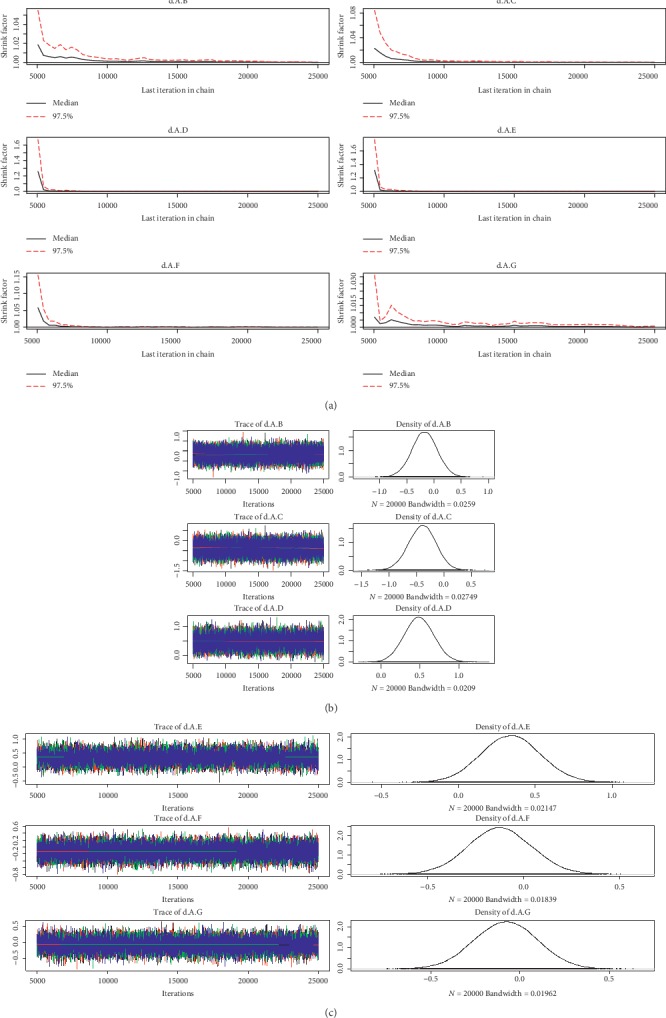
Brooks-Gelman-Rubin diagnostic plot and density plot for the efficacy of the comparison among different treatments of patients with normal renal function (A: warfarin; B: dabigatran110; C: dabigatran150; D: edoxaban30; E: edoxaban60; F:apixaban; G:rivaroxaban).

**Figure 3:**
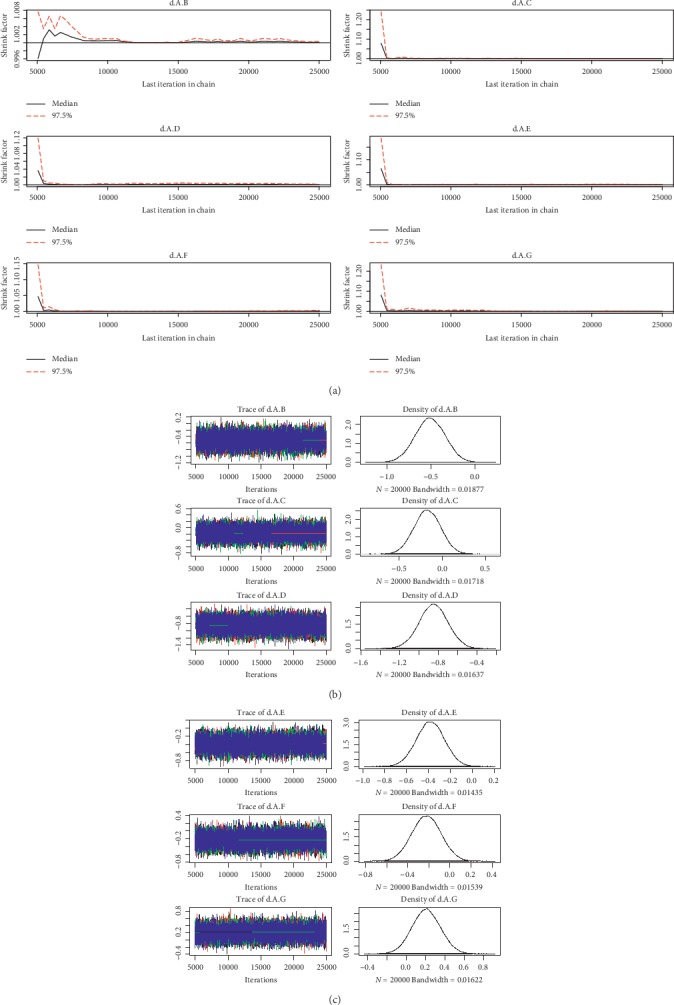
Brooks-Gelman-Rubin diagnostic plot and density plot for the safety of the comparison among different treatments of patients with normal renal function.

**Figure 4:**
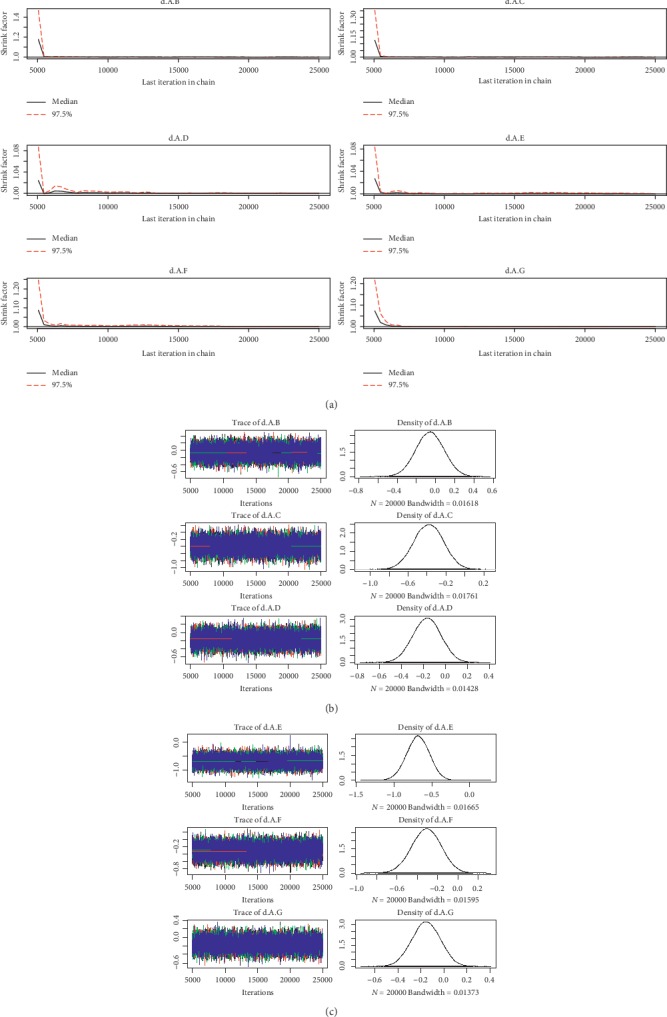
Brooks-Gelman-Rubin diagnostic plot and density plot for the efficacy of the comparison among different treatments of patients with mild renal function.

**Figure 5:**
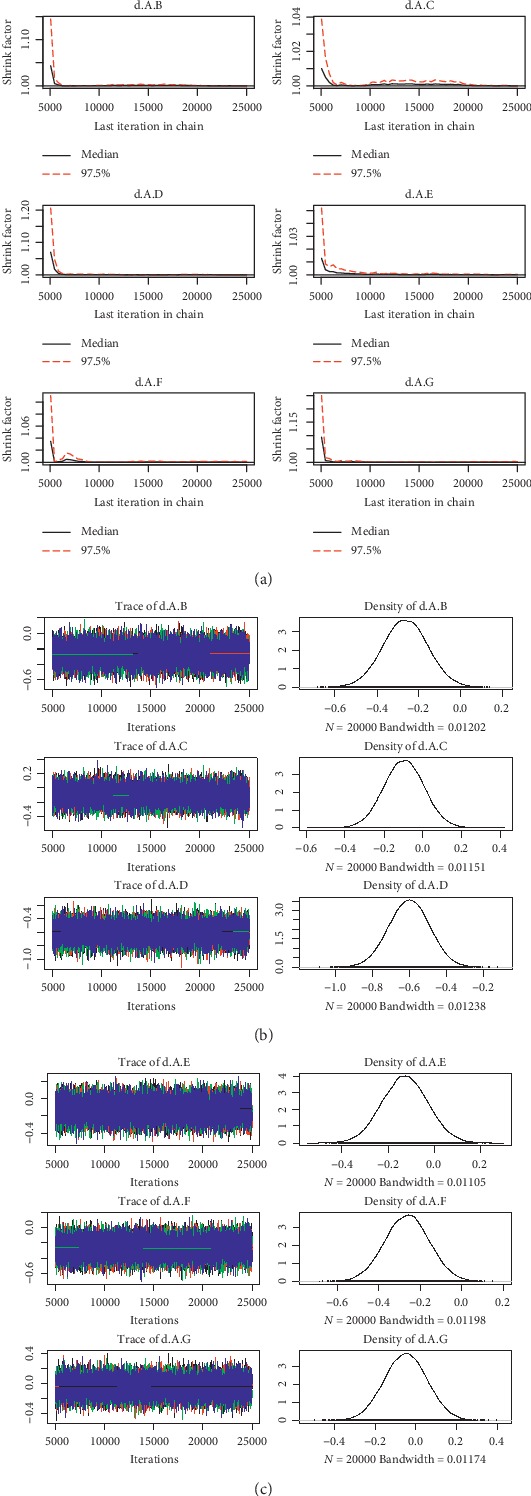
Brooks-Gelman-Rubin diagnostic plot and density plot for the safety of the comparison among different treatments of patients with mild renal function.

**Figure 6:**
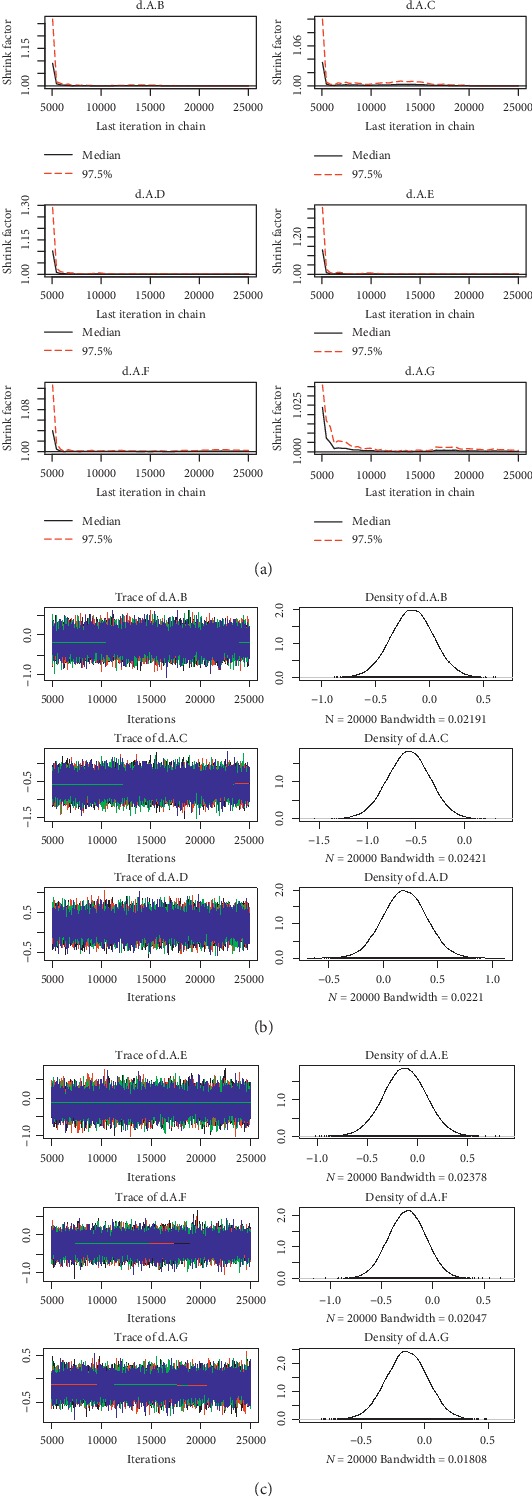
Brooks-Gelman-Rubin diagnostic plot and density plot for the efficacy of the comparison among different treatments of patients with moderate renal function.

**Figure 7:**
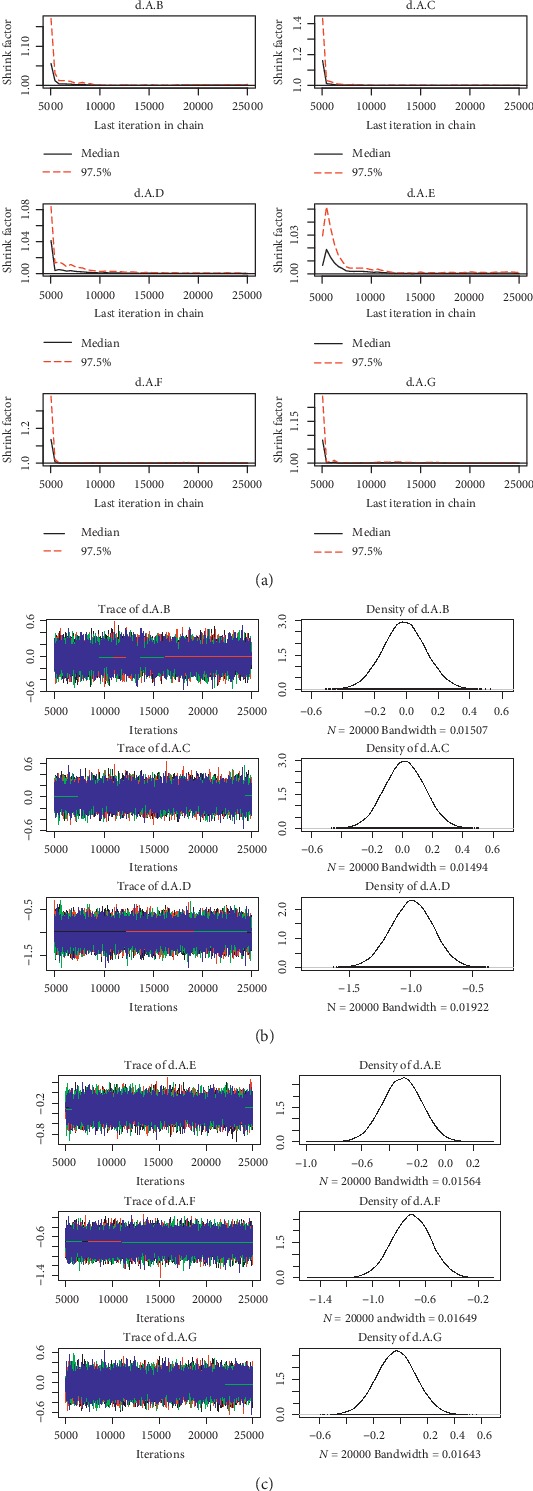
Brooks-Gelman-Rubin diagnostic plot and density plot for the safety of the comparison among different treatments of patients with moderate renal function.

For the convergence of our NMA, the Brooks-Gelman-Rubin diagnostic plot and density plot were obtained (Figures 2–7). We also calculated PSRF for each analysis and each result of PSRF was close to 1. According to the plots and PSRF, the model fitted better.

The pD and DIC were all calculated for both fixed and random-effects models, with the result showing that pD of each model was closed 10.0 and DIC was 20.0 for all analysis based on renal function, meaning there was no difference between fixed and random-effects models.

### 3.2. Subgroups of Patients with Normal Renal Function

**Figure 8:**
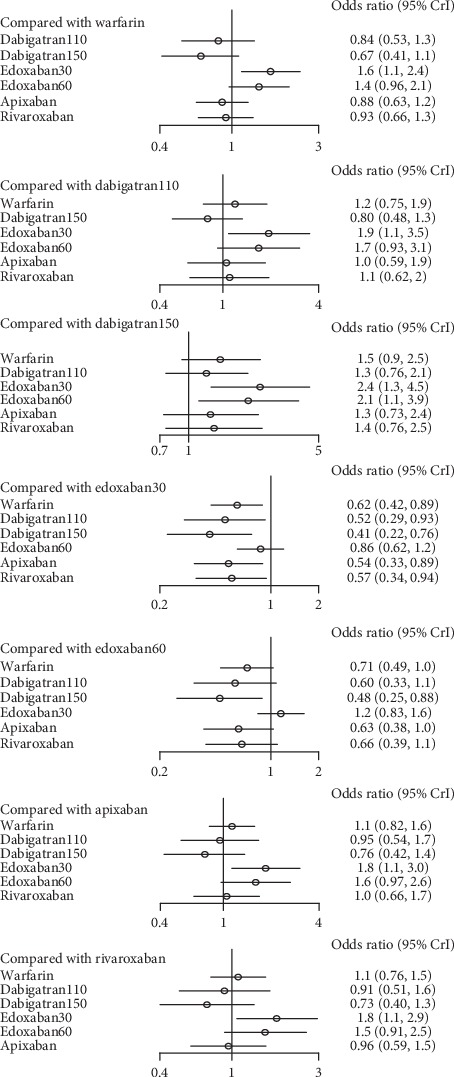
Ranking plots for the efficacy of the comparison among different treatments of patients with normal renal function.

**Figure 9:**
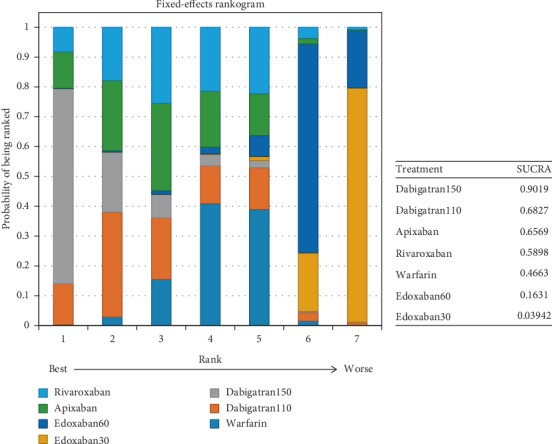
Ranking plots for the efficacy of the comparison among different treatments of patients with normal renal function (ranking indicates the probability to be the best treatment, the second best, the third best, and so on).

In comparison to edoxaban30, dabigatran110 (OR: 0.52, 95% CI: 0.29–0.93), dabigatran150 (0.41, 0.22–0.76), apixaban (0.54, 0.33–0.89), rivaroxaban (0.57, 0.34-0.94), and even warfarin (0.62, 0.42–0.89) were associated with a reduction of stroke or systemic embolism, but not edoxaban60. Besides that, dabigatran150 (0.48, 0.25–0.88) had lower rates relative to edoxaban60 in terms of stroke or systemic embolism. No difference was detected between edoxaban60 and edoxaban30 (Figure 8). (*τ*2 = 0.13, 95% CI 0.04–0.43) Dabigatran150 ranked as the most effective drug (SUCRA 0.90), followed by dabigatran110 (SUCRA 0.68), apixaban (SUCRA 0.66), and rivaroxaban (SUCRA 0.59) (Figure 9).

**Figure 10:**
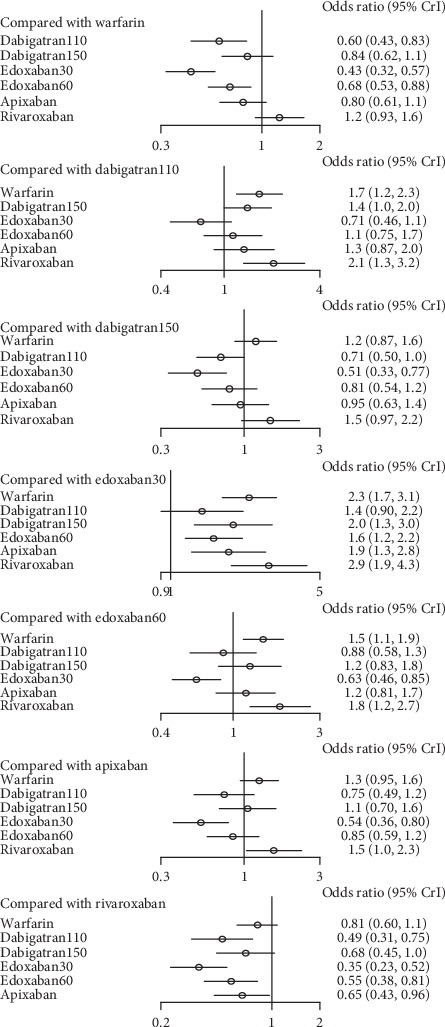
Forest plot for the safety of the comparison among different treatments of patients with normal renal function.

In contrast, the risk of major bleeding for patients treated with edoxaban30 was lower than dabigatran150 (0.51, 0.33–0.77), edoxaban60 (0.63, 0.46–0.85), apixaban (0.54, 0.36–0.80), and rivaroxaban (0.35, 0.23–0.52). In addition, dabigatran110 was associated with decreased risk of major bleeding compared to dabigatran150 (0.71, 0.5–1.0) and rivaroxaban (0.49, 0.31–0.75). Besides, edoxaban60 (0.55, 0.38–0.81) and apixaban (0.65, 0.43–0.96) reduced the risk of major bleeding contrast to rivaroxaban. But the difference between dabigatran110 and edoxaban30 was not significant. In comparison to warfarin, dabigatran110 (0.6, 0.43–0.83), edoxaban 30 (0.43, 0.32–0.57), and edoxaban60 (0.68, 0.53–0.88) were associated with relative risk reduction (Figure 10) (*τ*2 = 0.13, 0.04–0.44).

**Figure 11:**
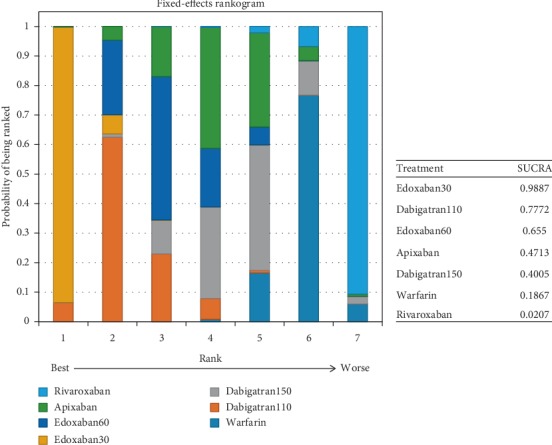
Ranking plots for the safety of the comparison among different treatments of patients with normal renal function.

There was high probability that edoxaban30 (SUCRA 0.99) ranked first for the preventing the major bleeding, in comparison to dabigatran110 (SUCRA 0.78) and edoxaban60 (SUCRA 0.66) (Figure 11).

### 3.3. Subgroup of Patients with Mild Renal Function

**Figure 12:**
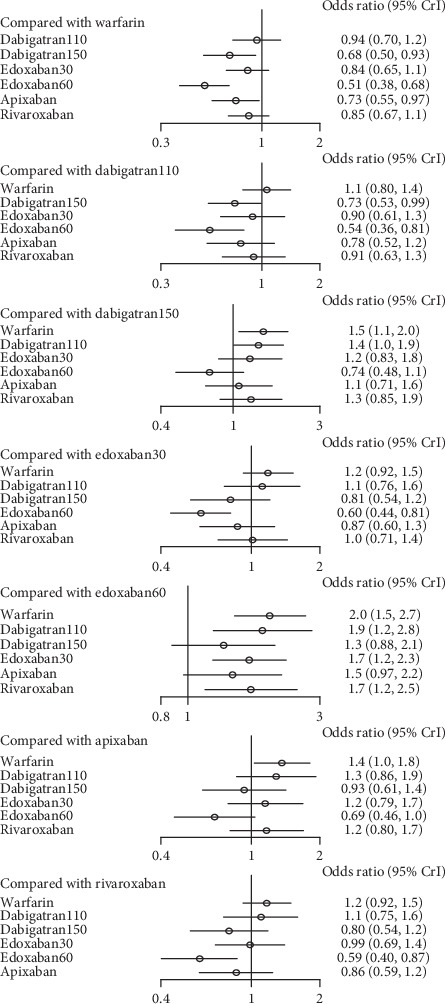
Forest plot for the efficacy of the comparison among different treatments of patients with mild renal function.

Dabigatran110 (1.9, 1.2–2.8), edoxaban30 (1.7, 1.2–2.3), and rivaroxaban (1.7, 1.2–2.5) took higher risk than edoxaban60 with respect to stroke or systemic embolism. In addition, dabigatran150 had lower risk than dabigatran110 (0.73, 0.53–0.99). In comparison to warfarin, dabigatran150 (0.68, 0.50–0.93), edoxaban60 (0.51, 0.380.68), and apixaban (0.73, 0.55–0.97) were more effective. There was no evidence to prove the difference among the NOACs left (Figure 12) (*τ*2 = 0.13, 0.04–0.44).

**Figure 13:**
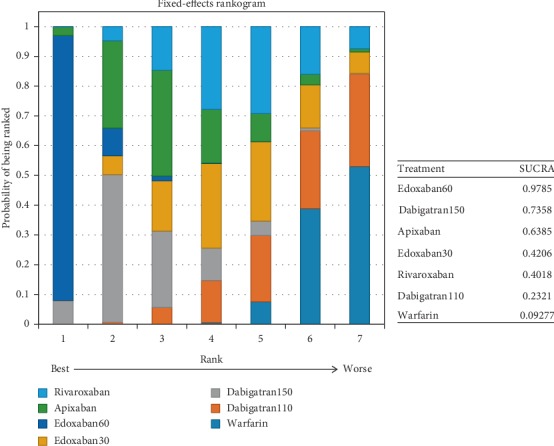
Ranking plots for the efficacy of the comparison among different treatments of patients with mild renal function.

With respect to the most effective drug for preventing stroke or systemic embolism, edoxaban60 ranked first (SUCRA 0.98), followed by dabigatran150 (SUCRA 0.74) and apixaban (SUCRA 0.64) (Figure 13).

**Figure 14:**
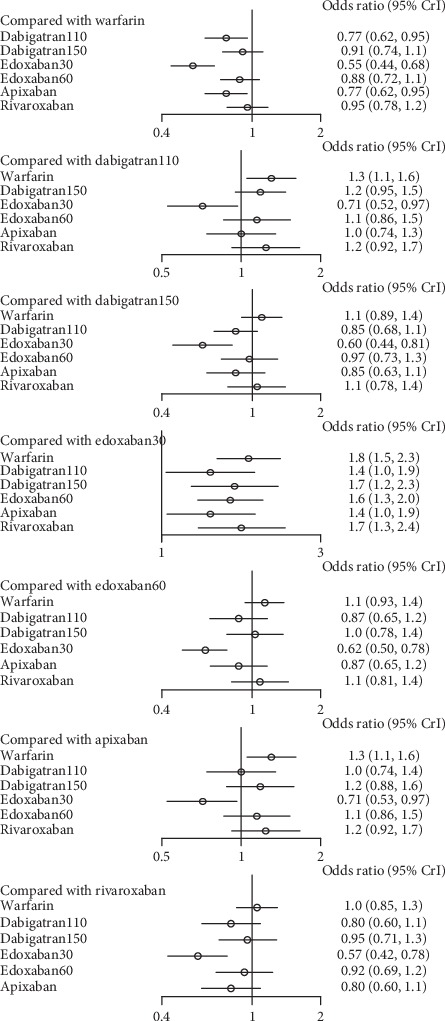
Forest plot for the safety of the comparison among different treatments of patients with mild renal function.

All the drugs had an increased rates of major bleeding in comparison to edoxaban30, including warfarin (1.8, 1.5–2.3), dabigatran110 (1.4, 1.0–1.9), dabigatran 150 (1.7, 1.2–2.3), edoxaban60 (1.6, 1.3–2.0), apixaban (1.4, 1.0–1.9), and rivaroxaban (1.7, 1.3–2.4). Compared to warfarin, dabigatran110 (0.77, 0.62–0.95) and apixaban (0.77, 0.62–0.95) took lower risk of major bleeding. In contrast, the difference among other interventions was not significant (Figure 14) (*τ*2 = 0.13, 0.04–0.42).

**Figure 15:**
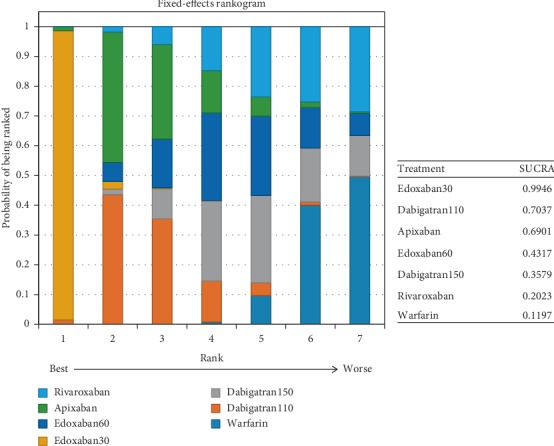
Ranking plots for the safety of the comparison among different treatments of patients with mild renal function.

Possibility of ranking first for the safest drug was edoxaban30 (SUCRA 0.99), followed by dabigatran110 (SUCRA 0.70) and apixaban (SUCRA 0.69) (Figure 15).

### 3.4. Subgroup of Patients with Moderate Renal Function

**Figure 16:**
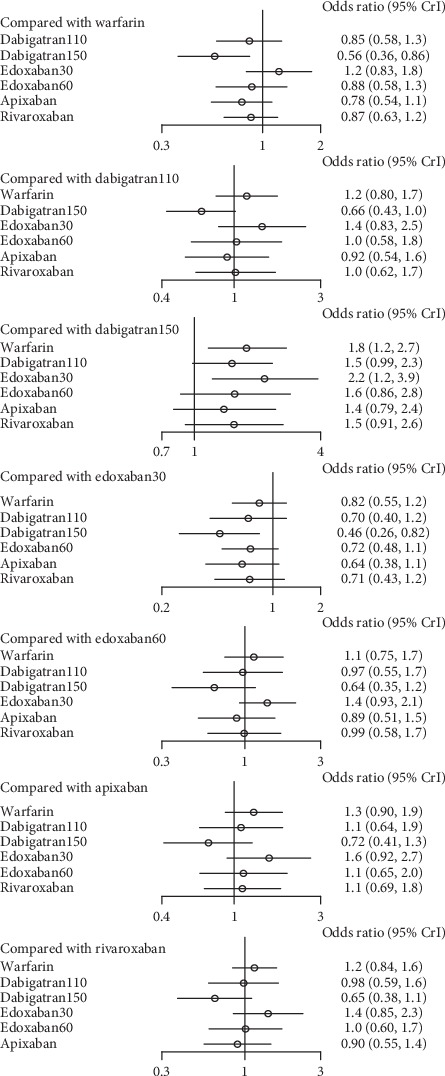
Forest plot for the efficacy of the comparison among different treatments of patients with moderate renal function.

**Figure 17:**
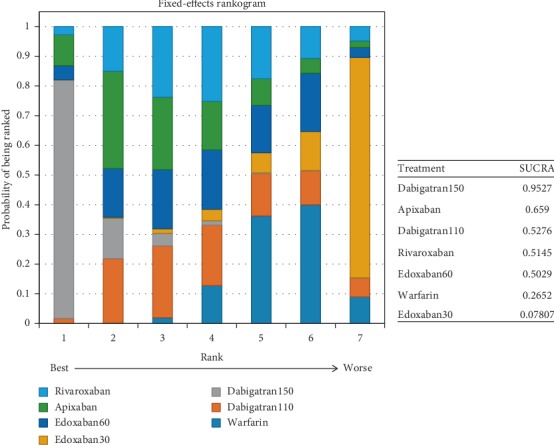
Ranking plots for the efficacy of the comparison among different treatments of patients with moderate renal function.

**Figure 18:**
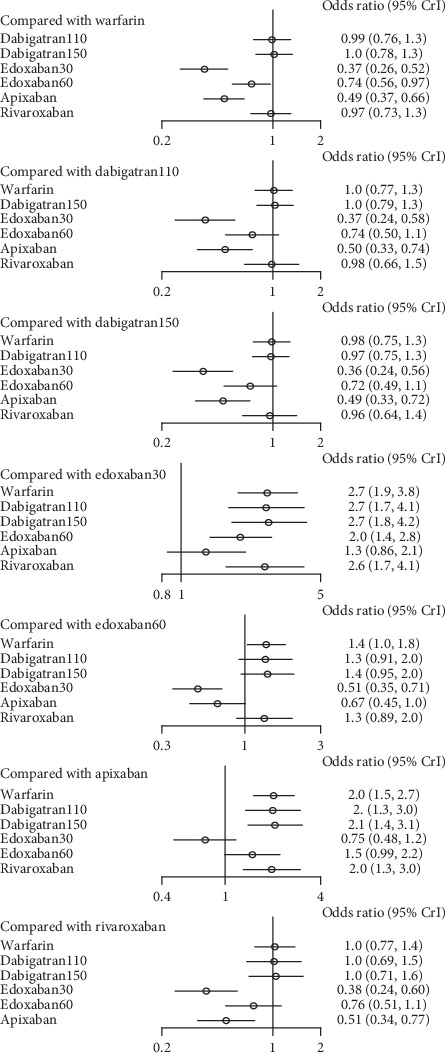
Forest plot for the safety of the comparison among different treatments of patients with moderate renal function.

In patients with moderate renal impairment, dabigatran150 reduced the rates of stroke or systemic embolism compared to warfarin (0.56, 0.36–0.86) and edoxaban30 (0.46, 0.26–0.82). There was no difference detected among other interventions (Figure 16) (*τ*2 = 0.13, 0.04–0.43). Dabigatran150 (SUCRA 0.95) ranked as the most effective drug compared to apixaban (SUCRA 0.66). Dabigatran110 (SUCRA 0.53), rivaroxaban (SUCRA 0.51), and edoxaban60 (SUCRA 0.50) had the similar probability of ranking third (Figure 17). Dabigatran110 (2.7, 1.7–4.1), dabigatran150 (2.7, 1.8–4.2), edoxaban60 (2.0, 1.4–2.8), and rivaroxaban (2.6, 1.7–4.1) were more dangerous for major bleeding than edxaban30. Besides, dabigatran110 (2.0, 1.3–3), dabigatran150 (2.1, 1.4–3.1), and rivaroxaban (2.0, 1.3–3) also increased the risk of major bleeding in contrast to apixaban. Compared to warfarin, edoxaban30 (0.37, 0.26–0.52), edoxaban60 (0.74, 0.56–0.97), and apixaban (0.49, 0.37–0.66) were associated with reduced risks of major bleeding (Figure 18) (*τ*2 = 0.13, 0.04–0.43).

**Figure 19:**
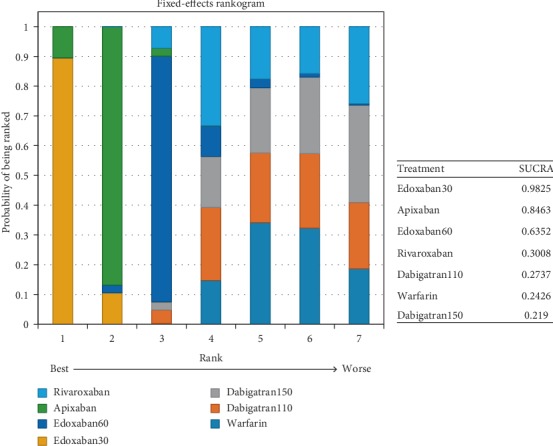
Ranking plots for the safety of the comparison among different treatments of patients with moderate renal function.

Probability of being the safest drug for preventing major bleeding was edoxaban30 (SUCRA 0.98), in comparison to apixaban (SUCRA 0.85) and edoxaban60 (SUCRA 0.64) (Figure 19).

## 4. Discussion

According to the recent studies published, renal function was an independent risk for stroke. In spite of numerous studies evaluating the effect of NOACs for preventing stroke or systemic embolism, few articles placed emphasis on the effects of NOACs for preventing stroke or systemic embolism and major bleeding in patients with different degrees of renal function, especially for head-to-head comparisons. With the aim to offer the better choice for patients with different levels of renal function, we identified 4 RCTs comparing the NOACs with respect to preventing stroke or systemic embolism and major bleeding in patients with different renal impairment in the NMA.

### 4.1. For Patients with Normal Renal Function

In patents with normal renal function, dabigatran150 (SUCRA 0.90) was the most effective drug for preventing stroke or systemic embolism in comparison to the edoxaban30 and edoxaban60, and dabigatran110 (SUCRA 0.68) was the second with effective prevention for stroke or systemic embolism, whereas edoxaban30 ranked as the least effective drug. When it referred to the safest drug, edoxaban30 (SUCRA 0.99) was associated with the great reduction of major bleeding compared to the other NOACs despite the least effective drug (SUCRA 0.04). In comparison, dabigatran110 (SUCRA 0.77) ranked second for preventing major bleeding and no significant difference was detected between edoxaban30 and dabigatran110. Despite dabigatran150 ranking as the most effective drug for preventing stroke or systemic embolism, the safety of such drug did not rank better (SUCRA 0.40). In comparison, dabigatran110 ranked second for preventing both stroke or systemic embolism (SUCRA 0.68) and major bleeding (SUCRA 0.77), meaning that dabigatran110 would be a better choice for patients with normal renal function.

A recent network meta-analysis published in 2018 compared different NOACs for their risk of ICH in patients with AF or venous thromboembolism (VTE), which drew a conclusion that dabigatran110 (SUCRA 0.83) ranked as the safest drug, but the NMA did not include the low dose edoxaban [27]. Another Bayesian meta-analysis published in 2015 also concluded that edoxaban30 was the safest drug with regard to preventing major bleeding, followed by dabigatran110 in patients with AF [28], which was closely consistent with our findings in patents with normal or mild renal function.

### 4.2. For Patients with Mild Renal Function

In patients with mild renal impairment, edoxaban60 (SUCRA 0.98) ranked first for the most effective drug in comparison to dabigatran110, edoxaban30, and rivaroxaban, while no significant difference was detected when compared to dabigatran150 or apixaban, with the former drug ranking second (SUCRA 0.74) and the latter ranking third (SUCRA 0.64). With respect to preventing major bleeding, edoxaban30 (SUCRA 0.99) ranked first as the most effective drug in comparison to the other NOACs. In comparison, dabigatran110 ranked second (SUCRA 0.70) and apixaban ranked third (SUCRA 0.69). Although edoxaban60 offered the greatest preventing stroke or systemic embolism, the influence of preventing major bleeding would not make it a better choice for its ranking (SUCRA 0.35). Also, edoxaban30 (SUCRA 0.42) could not offer a better prevention for stroke or systemic embolism in spite of the safest drug, and the same to the safety of dabigatran150 (SUCRA 0.35) and the efficacy of dabigatran110 (SUCRA 0.23). Compared to the other NOACs, apixaban would offer the better endings for both efficacy (SUCRA 0.64) and safety (SUCRA 0.69).

The article published by Katsanos et al. also gave the similar conclusion that apixaban would offer an optimal benefit in comparison to the other NOACs in terms of the efficacy and safety, which identified the four RCTs [29]. One meta-regression analysis also gave the findings that apixaban would offer a better safety profile for preventing major bleeding, as well as the effect of prevention stroke or systemic embolism in patients with mild renal impairment [30].

### 4.3. For Patients with Moderate Renal Function

For patients with moderate renal function, dabigatran150 (SUCRA 0.95) ranked first as the most effective drug in comparison to edoxaban30, while no difference was detected among other NOACs. In comparison, apixaban (SUCRA 0.66) ranked second, and dabigatran110 (SUCRA 0.53), rivaroxaban (SUCRA 0.51), and edoxaban60 (SUCRA 0.50) all could rank third. When it referred to preventing major bleeding, edoxaban30 (SUCRA 0.98) ranked as the safest drug in comparison to the other NOACs, but not apixaban, which ranked second (SUCRA 0.84), followed by edoxaban60 (SUCRA 0.64). Although dabigatran150 ranked first for preventing stroke or systemic embolism, it ranked as the least safe drug (SUCRA 0.21). And edoxaban (SUCRA 0.98) ranked as the least effective drug in spite of its best safety profile. In comparison, apixaban would be a better choice with respect to the efficacy (SUCRA 0.66) and safety (SUCRA 0.85). In addition, edoxaban60 offered better results for its efficacy (SUCRA 0.50) and safety (SUCRA 0.64).

Our findings were consistent with one network meta-analysis that apixaban and edoxaban60 would be a better choice in patients with moderate renal function [16] and one meta-regression analysis [30], which all include five RCTs.

Taking the characteristic of population into consideration, in comparison to the groups of patients treated with dabigatran150, edoxaban, apixaban, or rivaroxaban, patients associated with dabigatran110 were younger and had lower CHADS2 score, less rates of heart failure, and previous VKA use. However, the characteristic of patients with normal renal function treated with dabigatran110 was not available owing to some limitations. Even though, these data may give a possible explanation for the results, or meant there was a high tendency for such results achieved owing to these data. Besides, apixaban was associated with the least renal extraction (27%) [31], which may partly give an explanation for better results of patients treated with apixaban. In addition, we found that NOACs and warfarin would have an effect on the renal function, with different influence with respect to the various drugs. One article had compared the effects on renal function among NOACs and warfarin, finding that patients treated with NOACs were associated with renal function decline commonly, in spite of the lower risks of adverse renal outcomes of NOACs than warfarin [32]. However, the comparison of the effects associated with different NOACs on the renal function was unclear, meaning such effects may give a possible explanation for the results.

## 5. Limitation

Although the four RCTs identified in our analysis met the criterion better, the heterogeneity also existed as ROCKET, ARISTOTLE, and ENGAGE AF-TIMI 48 were double-blind trails, while RE-LY was open-labeled. In addition, the difference of criterion of inclusion, control of warfarin, and the definition of major bleeding should not be ignored. Besides, owing to the data collected, we could not form a closed loop for our NMA, which would have the effect on the examination of the model.

## 6. Conclusion

In patients with normal renal function, dabigatran 110 mg (bid) might have a better effect on the clinical results. And it does not coincide with patients taking dabigatran 110 mg with dose reduction for other factors including aged ≥75 years, renal impairment (CrCL 30–50 mL/min), gastritis, esophagitis, or gastroesophageal reflux, receiving concomitant verapamil, and so on. For patients with mild renal impairment, apixaban 5 mg (bid) would be a better choice for preventing stroke or systemic embolism and major bleeding, while apixaban 5 mg (bid) and edoxaban 60 mg (qd) were recommended for patients with moderate renal impairment. However, considering the fact of no RCTs for the head-to-head comparison, caution should be exercised over selecting each of NOACs for patients.

## Figures and Tables

**Table 1:** Search strategy.

Search terms	
#1	“Atrial fibrillation”: ti, ab, kw
#2	“Auricular fibrillation”: ti, ab, kw
#3	#1 or #2
#4	Dabigatran:ti, ab, kw
#5	apixaban: ti, ab, kw
#6	Rivaroxaban: ti, ab, kw
#7	Edoxaban: ti, ab, kw
#8	(Thrombin inhibit^*∗*^): ti, ab, kw
#9	(Factor next xa next inhibit^*∗*^): ti, ab, kw
#10	Or/#4-#9
#11	#3 and #10

**Table 2:** Assessment of risk of bias.

	Random sequence generation (selection bias)	Allocation concealment (selection bias)	Blinding of participants and personnel (performance bias)	Blinding of outcome assessment (detection bias)	Incomplete outcome data (attrition bias)	Selective reporting (reporting bias)	Other bias
RE-LY	Low	Low	Unclear	Low	Low	Low	Low
ARISTOTLE	Low	Low	Low	Low	Low	Low	Low
ROCKET-AF	Low	Low	Low	Low	Low	Low	Low
ENGAGE AF-TIMI 48	Low	Low	Low	Low	Low	Low	Low

**Table 3:** Characteristics of the included trials.

	RE-LY (18113)			ARISTOTLE (18201)		ROCKET-AF (14262)		ENGAGE AF 48 (21105)		
Dosing regiments	110 mg bid	150 mg bid	Adjusted	5 mg bid^(1)^	Adjusted	20 mg qd^(2)^	Adjusted	30 mg qd^(3)^	60 mg qd^(4)^	Adjusted
Median age, years	71.4	71.5	71.6	70	70	73	73	72	72	72

Type of AF, %										
Paroxysmal	32.1	32.6	33.8	15.1	15.5	17.5	17.8	25.3	24.9	26.1
Persistent, permanent	67.9	67.4	66.2	84.9	84.4	81.1	80.8	NR	NR	NR
Mean CHADS2 score	2.1	2.2	2.1	2.1	2.1	3.5	3.5	2.8	2.8	2.8
Previous stroke or TIA,%	19.9	20.3	19.8	19.2	19.7	54.9	54.6	28.3	28.1	28.5
Heart failure, %	32.2	31.8	31.9	35.5	35.4	62.6	62.3	57.5	58.2	56.6
Diabetes mellitus, %	23.4	23.1	23.4	25.0	24.9	40.4	39.5	35.8	36.4	36.2
Hypertension, %	78.8	78.9	78.9	87.3	87.6	90.3	90.8	93.6	93.7	93.3
Previous VKA use, %	50.1	50.2	48.6	51.7	57.2	62.3	62.5	58.8	58.8	59.2

Creatinine clearance, %										
Normal renal function, % >80 ml/min	32.3	32.0	32.2	41.2	41.4	32.3	31.3	NR	NR	NR
Mild renal function, % (50–80 ml/min)	48.6	48.1	48.5	41.6	41.8	46.6	46.8	NR	NR	NR
Moderate renal function, % (30–50 ml/min	19.4	19.2	19.4	16.5	16.6	21.0	20.6	19.3	19.6	19.0
Mean follow-up years	2.0	2.0	2.0	1.8	1.8	1.9	1.9	2.8	2.8	2.8

^(1)^2.5 mg bid of patients with two or more of the following criteria: ≥80 years, ≥60 kg, or serum creatinine ≥ 1.5 mg/dl. ^(2)^15 mg qd in patients with creatinine clearance: 30–49 ml/min. ^(3)^15 mg qd for patients with any of the following characteristics at the time of randomization or during the study: eGFR: 30–50 ml/min, weight ≤ 60 kg, or the concomitant use of verapamil or quinidine. ^(4)^30 mg qd for patients with any of the following characteristics at the time of randomization or during the study: eGFR: 30 to 50 ml/min, weight ≤ 60 kg, or the concomitant use of verapamil or quinidine.
